# Association of dietary intake of saturated fatty acids with obstructive sleep apnea: mediating effects of Life’s Crucial 9

**DOI:** 10.3389/fnut.2025.1503815

**Published:** 2025-02-17

**Authors:** Ruoyu Gou, Lili Chen, Zeyi Cheng, Jiawei Cun, Guanghua Li

**Affiliations:** ^1^School of Public Health, Ningxia Medical University, Yinchuan, Ningxia, China; ^2^Huadong Hospital Affiliated to Fudan University, Shanghai, China; ^3^Department of Cardiovascular Surgery, Ruijin Hospital, Shanghai Jiao Tong University School of Medicine, Shanghai, China; ^4^School of First Clinical Medical, Ningxia, Medical University, Yinchuan, Ningxia, China; ^5^School of Basic Medicine, Ningxia Medical University, Yinchuan, Ningxia, China

**Keywords:** saturated fatty acid (SFAs), obstructive sleep apnea (OSA), Life’s Crucial 9 (LC9), mediation, NHANES, cross-sectional study

## Abstract

**Introduction:**

Obstructive sleep apnea (OSA) is a global public health issue. Life’s Crucial 9 (LC9) is recognized as a powerful tool for assessing cardiovascular health. Although the etiology of OSA remains unclear, saturated fatty acids (SFAs) and cardiovascular health are increasingly regarded as a non-negligible element. This study aims to assess the association between dietary intake of SFAs and the risk of OSA, and the mediating effect of LC9.

**Methods:**

Based on the National Health and Nutrition Examination Survey (NHANES), dietary questionnaires of participant were collected, and the average values of 24-h dietary recall data over 2 days were obtained. A continuous cross-sectional analysis with dietary energy adjustment was employed. Weighted multivariable logistic regression models were used to estimate the weighted odds ratios (ORs) and their 95% confidence intervals (CIs) for SFAs and OSA. Evaluate the mediating role of LC9 in the relationship between SFAs and OSA.

**Results:**

A total of 13,563 participants aged 20 years and above were included in this study. The intakes of Sfa 4.0 and LC9 among participants with OSA were significantly lower than those in the normal population. After adjusting for confounding factors, total SFAs could increase the risk of OSA [Model 1, Q3, 0.03, 1.49 (1.03, 2.15); Model 2, Q3, 0.04, 1.47 (1.01, 2.13)]. It was emphasized that dietary intake of Sfa 12.0, Sfa 14.0, and Sfa 16.0 were protective factors for OSA, especially among participants aged 45–64 years and white individuals. Moreover, Sfa 12.0 exhibited a better protective effect in female participants [Q3, 0.04, 0.66 (0.45, 0.99)]. In addition, the cardiovascular health score - LC9 had a mediating effect in Sfa4.0 on OSA [Proportion of mediation: −0.035, 95% CI: (−0.058, −0.01); *p*= 0.002]. There was a nonlinear relationship between dietary intake of Sfa 12.0, Sfa 16.0, and Sfa 18.0 and OSA (*P-Nonlinear* = 0.013).

**Discussion:**

These findings suggest that dietary mixtures of saturated fatty acids increase the risk of OSA. Among them, SFA 4:0 can increase the risk of OSA through the level of cardiovascular health. However, contrary to traditional beliefs, long-chain saturated fatty acids can reduce the risk of OSA.

## 1 Introduction

Obstructive sleep apnea (OSA) is a common sleep disorder syndrome caused by upper airway obstruction during sleep in which patients repeatedly experience pauses in breathing or decreased airflow (1). OSA is associated with a variety of pathophysiological changes, such as sympathetic nervous system activation, decreased oxygen saturation in the blood, sleep arousal, and intermittent hypoxia (2). The severity of OSA is assessed using the apnea-hypopnea index (AHI), which is the number of episodes of apnea and hypopnea per hour of sleep (or recorded in a one-hour home test), with a higher AHI indicating a more severe form of OSA (3). Epidemiological studies have shown that 425 million adults (30–69 years old) worldwide suffer from OSA [1]. The prevalence of OSA is higher in patients with cardiovascular diseases: arrhythmia (50–20%), heart failure (55–12%), hypertension (83% mild, 30% moderate to severe), stroke (75–57%), and coronary heart disease (65–38%) (4). Furthermore, the prevalence of OSA is higher in the hypertension cohort, with a higher prevalence among patients with resistant hypertension (5). The etiology of OSA is unclear due to complex environmental factors and is becoming a public health problem worldwide.

Chronic intermittent hypoxia is a critical pathophysiological mechanism in obstructive sleep apnea (OSA), and it is associated with a range of pathological functional disorders (6). These include sympathetic nervous system activation, systemic inflammatory responses, and dyslipidemia, with the latter’s pathogenesis being closely linked to both cardiovascular disease and OSA (7). Dyslipidemia is characterized by abnormal levels of total cholesterol, triglycerides, low-density lipoprotein (LDL) cholesterol, and high-density lipoprotein (HDL) cholesterol, all of which are considered significant risk factors for vascular diseases (8, 9). Research has demonstrated that OSA is associated with impaired lipid metabolism (10). Furthermore, poor sleep quality is often accompanied by poor diet quality, and these dietary factors interact with sleep patterns to influence the risk of cardiovascular disease (CVD) (11). The cholesterol/saturated fat index (CSI) is strongly correlated with OSA, with lower CSI levels indicating reduced levels of cholesterol and saturated fatty acids (7). Saturated fatty acids (SFAs), which lack unsaturated double bonds, are a crucial component of lipids (12). Common sources of SFAs include coconut oil, rice bran oil, red meat, high-fat dairy products, and human breast milk. SFAs can be categorized into short-chain fatty acids (SCFAs) with fewer than 6 carbon atoms (e.g., SFA 4:0), medium-chain fatty acids (MCFAs) with 6–12 carbon atoms (e.g., SFA 6:0, SFA 8:0, SFA 10:0, SFA 12:0), and long-chain fatty acids (LCFAs) with 12 or more carbon atoms (e.g., SFA 14:0, SFA 16:0, SFA 18:0). Epidemiological studies have shown that increased daily intake of SFAs is associated with reduced sleep duration (13) and greater severity of OSA in overweight individuals (14). Animal studies have observed that mice on a high-fat diet exhibit an increased frequency of rapid eye movement (REM) sleep episodes during the dark phase and a decreased number of awakenings (15).

Higher concentrations of acetate, butyrate and propionate were associated with lower sleep efficiency and longer sleep latency, suggesting that short-chain fatty acids may influence sleep continuity (16). Caprylic acid, in particular, may affect sleep indirectly by influencing metabolic pathways (energy supply and gut microbiota) (17). Therefore, we hypothesized that dietary SFAs may increase the risk of developing OSA. And we further explored the effects of multiple subtypes on OSA.

A substantial body of research supports the link between irregular sleep patterns and cardiovascular disease (CVD) (18) The prevailing view posits that poor sleep quality is a risk factor for cardiovascular disease, leading researchers to investigate poor sleep states as a potential cause. However, the Turkish Collaboration of Sleep Apnea Cardiovascular Trialists (TURCOSACT) has highlighted that the relationship between OSA and traditionally recognized cardiovascular diseases is bidirectional (19). The American Heart Association’s Life’s Essential 8 (LE8) cardiovascular scoring system, designed to assess participants’ cardiovascular health at the time of the survey and calculate the LE8 score for each indicator, has been previously published (20). Chinese scholars have since updated the LE8 scoring system, proposing the Life’s Crucial 9 (LC9) cardiovascular health scoring system, which underscores the importance of mental health in preventing CVD (21). LC9 offers a more comprehensive evaluation of cardiovascular health (21). Patients with cardiovascular disease may also experience OSA, and effectively treating cardiovascular disease can potentially improve obstructive sleep apnea (19). For instance, the prevalence of OSA is notably higher in the CVD population, and CVD itself may contribute to the onset or exacerbation of OSA (19). Additionally, research has identified elevated systolic blood pressure (SBP) and diastolic blood pressure (DBP) as independent risk factors for OSA (22). Consequently, we hypothesize that poor cardiovascular health may serve as a risk factor for OSA. The 2013 report by the American Heart Association and the American College of Cardiology (AHA/ACC) on lifestyle management guidelines for reducing cardiovascular risk provides robust evidence that reducing saturated fatty acid (SFA) intake (to 5–6% of total calories) can lower LDL cholesterol (23). Extensive research supports the association between SFA intake and an increased risk of cardiovascular disease (24, 25). Prior studies have also identified age, gender, and race as established risk factors for OSA (26, 27), with significant variations in disease prevalence across different subgroups. In conclusion, dietary intake of SFAs may increase the risk of OSA, potentially mediated by its impact on cardiovascular health.

## 2 Materials and methods

### 2.1 Study populations

**Figure 1:**
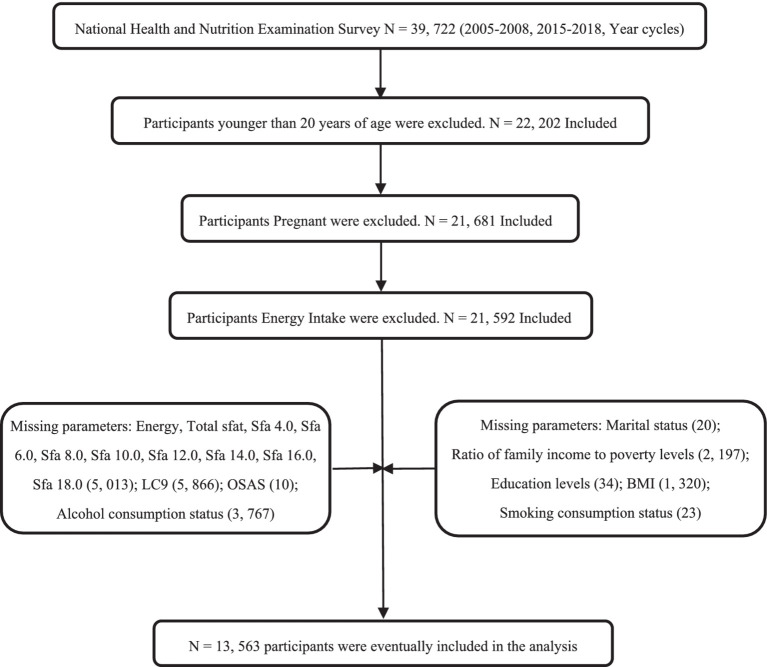
Flow chart of the screening process for the selection of the study population.

The National Health and Nutrition Examination Survey (NHANES) is an ongoing survey conducted by the National Center for Health Statistics (NCHS), a division of the Centers for Disease Control and Prevention (CDC). The primary objective of NHANES is to assess the health and nutritional status of the United States population. The survey employs a complex multistage probabilistic cluster sampling design, with approximately 5,000 participants each year. Data collected encompass demographic information, anthropometric measurements, laboratory test results, and dietary data. Prior to participation, all subjects provide written informed consent for the use of their data in health-related statistical research. Comprehensive program details, data collection protocols, and available datasets are publicly accessible at http://www.cdc.gov/nchs/nhanes.html. This study analyzed NHANES survey cycles from 2005 to 2008 and 2015 to 2018, focusing on subjects aged 20 years and older with complete data. A total of 13,563 subjects were included in the analysis, representing the general population of 15,519,308 individuals in the United States. As shown in Figure 1.

### 2.2 Assessment of nutrients

Dietary sources of energy, total saturated fatty acids (SFA), and their subtypes (SFA 4:0, SFA 6:0, SFA 8:0, SFA 10:0, SFA 12:0, SFA 14:0, SFA 16:0, and SFA 18:0) were obtained through two 24-h recall interviews. The selection and calculation of nutrient types were based on the NHANES support materials available at the following URL: https://wwwn.cdc.gov/Nchs/Nhanes/2017-2018/DSQTOT_J.htm. The measurement guide (28) provided detailed information about the instrument used to measure the dietary intake of respondents. Dietary nutrient intakes were calculated using the Food and Nutrient Database for Dietary Studies 5.0 (FNDDS 5.0) standard of the United States Department of Agriculture (29, 30). This approach ensures that the dietary intake data collected from the respondents are accurately analyzed and provide valuable insights into the sources of energy and saturated fatty acids in their diets.

### 2.3 Assessment of OSA (outcome)

Obstructive Sleep Apnea (OSA) Symptoms: OSA is diagnosed based on responses to three dichotomous questions. These questions include: (1) Snoring three or more nights per week; (2) Snorting, gasping, or stopping breathing three or more nights per week; and (3) Experiencing excessive daytime sleepiness 16–30 times per month, despite sleeping seven or more hours per night on weekdays or work nights (31). Individuals are classified as positive for OSA if they answer “yes” to at least one of these three questions.

### 2.4 Measurement of cardiovascular health score

In previous studies, the AHA proposed the LE8 score to assess cardiovascular health status (20). The LE8 score consists of four health behaviors (diet, physical activity, nicotine exposure, and sleep duration) and four health factors (body mass index, non-high-density lipoprotein cholesterol, blood glucose, and blood pressure). It has been reported that considering mental health factors is the best and most equitable basis for achieving CVH (32). Among mental health factors, depression has received significant attention (32). Depression is an independent non-traditional risk factor for cardiovascular disease (CVD) (33). In NHANES, a higher PHQ-9 score indicates a higher level of current depressive symptoms. Depression scores were designated as 100, 75, 50, 25, and 0, corresponding to PHQ-9 scores of 0–4, 5–9, 10–14, 15–19, and 20–27 (34). The LC9 score was calculated as the average of the eight indicators in the LE8 score and the depression score (34). Before this, the AHA only proposed the concept of constructing the LC9 scoring system but did not officially release the composition and calculation method of the LC9 indicators (32). The latest research has proposed the construction process and calculation method of the LC9 scoring system (21) and verified the association between LC9 and cardiovascular mortality and all-cause mortality, which makes the cardiovascular health risk scoring system more complete and provides a direction for future research (21).

### 2.5 Defining covariates

Covariates were defined as patient age, sex, ethnicity/race, marital status, family income-to-poverty ratio, education status, BMI, smoking status, and alcohol consumption status. Race was classified as: White, Black, Mexican, and Other. Education status was described as: Less than 11th grade, High school graduate, College graduate or above. Family income-to-poverty ratio was categorized into four groups based on a scale from 1.3 to >5: 1.3, 1.3–3, 3–5, and >5. A higher ratio indicated a higher income status. Marital status was classified as: Married, Divorced, and Unmarried. Alcohol consumption frequency and the sex of the consumer were recorded, and alcohol consumption was defined as: Never, Former, Mild, Moderate, and Heavy. Smoking status was categorized as: Currently smoking, formerly smoking, or Never smoking. Never smokers were defined as those who had smoked fewer than 100 cigarettes in their lifetime, former smokers as those who had smoked more than 100 cigarettes but were no longer smoking, and current smokers as those who had smoked more than 100 cigarettes consistently or inconsistently. BMI was defined as: <25 kg/m^2^, 25–30 kg/m^2^, and >30 kg/m^2^.

### 2.6 Statistical analysis

We employed the NHANES criteria for statistical analysis (oversampling, stratification, and clustering) to ensure that the appropriate number of U.S. adults to use in this study. In addition, we evaluated the required statistical tests for weight adjustment. Categorical variables were assessed via chi-squared test.

Nutrient intake was corrected using the residual method. Nutrient intakes were grouped into quartiles. The lowest quartile (Q1) was defined as the reference group in each model. We employed logistic regression models to assess relationships between SFAs and their subtypes (Sfas 4:0, Sfas 6:0, Sfas 8:0, Sfas 10:0, Sfas 12:0, Sfas 14:0, Sfas 16:0, and Sfas 18:0) intake with OSA. The acquired data are presented as weighted odds ratios (OR) with corresponding 95% confidence intervals (CI) (OR [95% CI]). Stratified analyses were conducted for sex, age, ethnicity, using weighted multivariate logistic regression. Three models were developed:(1) Crude model, unadjusted model; (2) Model 1, a model adjusted for age, sex, and race or ethnicity;(3) Model 2, a model adjusted for age, sex, race or ethnicity, education level, marital status, LC9, Smoking consumption status, Alcohol consumption status, BMI and Ratio of family income to poverty levels. We generated restricted cubic spline (RCS) plots to display patterns in variables of significance in logistic regression. Using the RCS plots, we determined the presence or absence of a nonlinear association between the mentioned exposure factors and OSA. The potential mediating role of LC9 in the association between SFAs and OSA risk was estimated using a parallel mediation model implemented in the R package “mediation.” Mediation analyses were conducted using a quasi-Bayesian Monte Carlo approach with 1,000 simulations based on normal approximation. The direct effect (DE) indicates the impact of SFAs exposure on OSA in the absence of a mediator, while the indirect effect (IE) reflects the influence of LC9 exposure on OSA through mediators. The proportion of mediation was calculated as the ratio of IE to the total effect (TE). R 4.2.2 (R Project for Statistical Computing) were used to conduct the analyses. Significance was set at two-sided *p* < 0.05.

## 3 Results

### 3.1 Characteristics of the study population

This study included 13,563 participants (9,470 non-OSA participants and 4,093 OSA participants), and their demographic characteristics are presented in Table 1. Compared with non-OSA participants, OSA participants had lower intakes of SFA 4.0 and SFA 8.0, with means of 0.52 (0.01) and 0.25 (0.00), respectively. OSA participants had a higher intake of SFA 18.0, 6.59 (0.04), compared to non-OSA participants, 6.48 (0.04). Additionally, OSA participants had lower LC9 scores, 65.29 (0.42), compared with non-OSA participants. Statistical analysis revealed significant differences in demographic characteristics between non-OSA and OSA participants, including age, sex, marital status, smoking status, alcohol consumption status, and BMI (*p*-value <0.001). However, no significant differences were found between the two groups in terms of ethnicity/race, family income-to-poverty ratio, and education level (*p* > 0.05).

**Table 1:** Characteristics of participants with and without OSA.

Parameter	No. of participants [Weighted %; mean (SE)]			
	All participants (*N* = 13, 563)	Non-OSA (*N* = 9, 470)	OSA (N = 4, 093)	*P*-value^a^
Total sfat	26.27 (0.11)	26.21 (0.12)	26.39 (0.17)	0.38
Sfa 4.0	0.53 (0.01)	0.54 (0.01)	0.52 (0.01)	0.01
Sfa 6.0	0.31 (0.00)	0.31 (0.00)	0.30 (0.01)	0.06
Sfa 8.0	0.26 (0.00)	0.26 (0.00)	0.25 (0.00)	0.02
Sfa 10.0	0.49 (0.00)	0.49 (0.00)	0.48 (0.01)	0.08
Sfa 12.0	0.84 (0.01)	0.86 (0.01)	0.82 (0.02)	0.17
Sfa 14.0	2.23 (0.02)	2.25 (0.02)	2.20 (0.03)	0.13
Sfa 16.0	14.19 (0.05)	14.14 (0.06)	14.31 (0.09)	0.11
Sfa 18.0	6.52 (0.03)	6.48 (0.04)	6.59 (0.04)	0.04
Life’s Crucial 9 (LC9)	69.60 (0.34)	71.50 (0.34)	65.29 (0.42)	< 0.001
Age				< 0.001
20–44	5,505 (44.54)	4,008 (46.58)	1,497 (39.89)	
45–64	4,753 (37.09)	3,065 (34.77)	1,688 (42.36)	
> = 65	3,305 (18.38)	2,397 (18.65)	908 (17.76)	
Sex				< 0.001
Female	6,896 (51.75)	5,022 (54.62)	1874 (45.20)	
Male	6,667 (48.25)	4,448 (45.38)	2,219 (54.80)	
Ethnic/race				0.66
White people	6,140 (71.02)	4,255 (70.74)	1885 (71.67)	
Black people	2,894 (10.33)	2032 (10.38)	862 (10.23)	
Mexican people	2,140 (7.38)	1,524 (7.54)	616 (7.01)	
Other	2,389 (11.26)	1,659 (11.34)	730 (11.08)	
Marital				< 0.001
Married	8,374 (65.62)	5,659 (63.62)	2,715 (70.19)	
Never married	2,982 (18.15)	2,132 (18.40)	850 (17.56)	
Separated	2,207 (16.23)	1,679 (17.98)	528 (12.25)	
Ratio of family income to poverty levels				0.58
<1.3	3,707 (17.65)	2,603 (17.70)	1,104 (17.53)	
1.3–3	4,575 (29.18)	3,156 (28.69)	1,419 (30.30)	
3–5	2,717 (25.13)	1900 (25.45)	817 (24.42)	
≥5	2,564 (28.03)	1811 (28.16)	753 (27.75)	
Education levels				0.19
Less than 11th grade	3,034 (13.48)	2,145 (13.44)	889 (13.57)	
High school graduate	6,446 (54.97)	4,562 (55.59)	1884 (53.58)	
College graduate or above	4,083 (31.55)	2,763 (30.98)	1,320 (32.86)	
Smoking consumption status				< 0.001
Former	3,468 (25.93)	2,321 (24.93)	1,147 (28.21)	
Never	7,364 (54.51)	5,389 (57.16)	1975 (48.46)	
Now	2,731 (19.57)	1760 (17.91)	971 (23.34)	
Alcohol consumption status				< 0.001
Former	2,142 (12.56)	1,476 (11.87)	666 (14.14)	
Heavy	2,589 (20.33)	1756 (19.61)	833 (21.97)	
Mild	4,800 (38.65)	3,292 (38.55)	1,508 (38.88)	
Moderate	2,201 (18.23)	1,527 (18.36)	674 (17.94)	
Never	1831 (10.22)	1,419 (11.60)	412 (7.07)	
BMI				< 0.001
<25, kg/m^2^	3,708 (29.12)	3,035 (34.55)	673 (16.74)	
25–30, kg/m^2^	4,496 (32.23)	3,222 (32.99)	1,274 (30.48)	
≥30, kg/m^2^	5,359 (38.65)	3,213 (32.46)	2,146 (52.78)	

^aT-test for continuous variables and Rao-Scott χ2 test for categorical variables.^

### 3.2 Univariate logistic regression results (SFAs and OSA; SFAs and LC9; SFAs and LC9)

In the weighted univariate logistic regression, after adjusting for potential confounding factors, SFAs were significantly associated with OSA. We reported the statistically significant quantile levels, *p*-values and OR (95% CI) of the association between SFAs and OSA. Compared with Q1, Total sfat [Q4, 0.003, 0.80 (0.69, 0.92)], Sfa 4.0 [Q3, 0.024, 0.86 (0.76, 0.98); Q4, 0.02, 0.86 (0.77, 0.98)], Sfa 6.0 [Q3, 0.044, 0.871 (0.76, 0.99)], Sfa 8.0 [Q4, 0.021, 0.84 (0.73, 0.97)], Sfa 10.0 [Q4, 0.027, 0.86 (0.76, 0.98)], Sfa 12.0 [Q2, 0.049, 0.87 (0.75, 1.00); Q3, 0.006, 0.81 (0.70, 0.94); Q4, 0.004, 0.81 (0.71, 0.93)], Sfa 14.0 [Q2, 0.042, 0.87 (0.75, 0.99); Q4, <0.001, 0.78 (0.69, 0.89)], Sfa 16.0 [Q2, 0.002, 0.80 (0.70, 0.91); Q3, 0.011, 0.81 (0.70, 0.95); Q4, 0.008, 0.83 (0.73, 0.95)], Sfa 18.0 [Q2, 0.053, 0.87 (0.75, 1.00); Q4, 0.01, 0.82 (0.71, 0.95)]. As shown in Table 2.

**Table 2:** Univariate logistic regression results of saturated fatty acids and OSA.

Parameter		Estimate	*P*-value	OR (95% CI)
Total sfat	[−17.16, 21.15]	ref	ref	ref
	(21.15, 25.31]	−0.22	0.007	0.81 (0.69, 0.94)
	(25.31, 29.66]	−0.11	0.206	0.90 (0.76, 1.06)
	(29.66, 75.86]	−0.23	0.003	0.80 (0.69, 0.92)
Sfa 4.0	[−0.92, 0.28]	ref	ref	ref
	(0.28, 0.45]	−0.1	0.159	0.90 (0.78, 1.04)
	(0.45, 0.66]	−0.15	0.024	0.86 (0.76, 0.98)
	(0.66, 4.18]	−0.15	0.02	0.86 (0.77, 0.98)
Sfa 6.0	[−0.49, 0.16]	ref	ref	ref
	(0.16, 0.26]	−0.11	0.145	0.90 (0.77, 1.04)
	(0.26, 0.38]	−0.14	0.044	0.871 (0.76, 0.99)
	(0.38, 2.45]	−0.13	0.058	0.875 (0.76, 1.01)
Sfa 8.0	[−0.35, 0.14]	ref	ref	ref
	(0.14, 0.21]	−0.06	0.449	0.95 (0.82, 1.10)
	(0.21, 0.31]	−0.12	0.119	0.89 (0.76, 1.03)
	(0.31, 3.62]	−0.17	0.021	0.84 (0.73, 0.97)
Sfa 10.0	[−0.65, 0.27]	ref	ref	ref
	(0.27, 0.41]	−0.08	0.201	0.92 (0.81, 1.05)
	(0.41, 0.59]	−0.12	0.105	0.88 (0.76, 1.03)
	(0.59, 3.28]	−0.15	0.027	0.86 (0.76, 0.98)
Sfa 12.0	[−1.03, 0.37]	ref	ref	ref
	(0.37, 0.59]	−0.14	0.049	0.87 (0.75, 1.00)
	(0.59, 0.89]	−0.21	0.006	0.81 (0.70, 0.94)
	(0.89, 22.92]	−0.21	0.004	0.81 (0.71, 0.93)
Sfa 14.0	[−2.36, 1.42]	ref	ref	ref
	(1.42, 1.99]	−0.14	0.042	0.87 (0.75, 0.99)
	(1.99, 2.65]	−0.15	0.059	0.86 (0.74, 1.01)
	(2.65, 12.57]	−0.25	<0.001	0.78 (0.69, 0.89)
	[−8.32, 11.83]	ref	ref	ref
Sfa 16.0	(11.83, 13.88]	−0.23	0.002	0.80 (0.70, 0.91)
	(13.88, 16.02]	−0.21	0.011	0.81 (0.70, 0.95)
	(16.02, 41.30]	−0.18	0.008	0.83 (0.73, 0.95)
Sfa 18.0	[−4.20, 5.27]	ref	ref	ref
	(5.27, 6.35]	−0.14	0.053	0.87 (0.75, 1.00)
	(6.35, 7.50]	−0.15	0.075	0.87 (0.74, 1.02)
	(7.50, 19.08]	−0.2	0.01	0.82 (0.71, 0.95)

Model Adjusted for sex, age, ethnic/race, marital, family income-to-poverty ratio, Education levels, LC9, BMI, alcohol consumption status, and smoking consumption status.

In the weighted univariate logistic regression, after adjusting for potential confounding factors, SFAs and LC9 were significantly correlated. We reported the statistically significant quantile levels, Estimate, *p*-value and OR (95% CI) of the association between SFAs and LC9. Compared with Q1, Total sfat [Q2, −1, 0.003, −1 (−1.62, −0.37); Q3, −2.59, <0.001, −2.59 (−3.25, −1.93); Q4, −3.44, <0.001, −3.44 (−4.11, −2.77)], Sfa 4.0 [Q4, −1.98, <0.001, −1.98 (−2.65, −1.30)], Sfa 6.0 [Q4, −1.2, 0.002, −1.2 (−1.93, −0.46)], Sfa 8.0 [Q4, −1.2, <0.001, −1.2 (−1.86, −0.55)], Sfa 10.0 [Q4, −1.21, <0.001, −1.21 (−1.89, −0.52)], Sfa 12.0 [Q4, −1.07, 0.003, −1.07 (−1.75, −0.39)], Sfa 14.0 [Q3, −1.3, <0.001, −1.3 (−1.91, −0.68); Q4, −2.39, <0.001, −2.39 (−3.11, −1.67)], Sfa 16.0 [Q2, −1.05, 0.003, −1.05 (−1.72, −0.37); Q3, −2.42, <0.001, −2.42 (−3.15, −1.69); Q4, −3.2, <0.001, −3.2 (−3.86, −2.54)], Sfa 18.0 [Q2, −1.97, <0.001, −1.97 (−2.61, −1.33); Q3, −3.28, <0.001, −3.28 (−3.92, −2.64); Q4, −4.43, <0.001, −4.43 (−5.05, −3.81)]. As shown in Table 3.

**Table 3:** Multivariate logistic regression results of SFAs and LC9.

Parameter		Estimate	*P*-value	OR (95% CI)
Total sfat	[−17.16, 21.15]	Ref	Ref	Ref
	(21.15, 25.31]	−1	0.003	−1 (−1.62, −0.37)
	(25.31, 29.66]	−2.59	<0.001	−2.59 (−3.25, −1.93)
	(29.66, 75.86]	−3.44	<0.001	−3.44 (−4.11, −2.77)
Sfa 4.0	[−0.92, 0.28]	ref	ref	ref
	(0.28, 0.45]	−0.02	0.94	−0.02 (−0.71, 0.66)
	(0.45, 0.66]	−0.49	0.24	−0.49 (−1.33, 0.35)
	(0.66, 4.18]	−1.98	<0.001	−1.98 (−2.65, −1.30)
Sfa 6.0	[−0.49, 0.16]	ref	ref	ref
	(0.16, 0.26]	0.35	0.34	0.35 (−0.38, 1.07)
	(0.26, 0.38]	0.08	0.8	0.08 (−0.59, 0.76)
	(0.38, 2.45]	−1.2	0.002	−1.2 (−1.93, −0.46)
Sfa 8.0	[−0.35, 0.14]	ref	ref	ref
	(0.14, 0.21]	0	0.99	0 (−0.67, 0.66)
	(0.21, 0.31]	−0.51	0.13	−0.51 (−1.18, 0.16)
	(0.31, 3.62]	−1.2	<0.001	−1.2 (−1.86, −0.55)
Sfa 10.0	[−0.65, 0.27]	ref	ref	ref
	(0.27, 0.41]	−0.19	0.53	−0.19 (−0.80, 0.42)
	(0.41, 0.59]	−0.35	0.29	−0.35 (−1.01, 0.31)
	(0.59, 3.28]	−1.21	<0.001	−1.21 (−1.89, −0.52)
Sfa 12.0	[−1.03, 0.37]	ref	ref	ref
	(0.37, 0.59]	−0.08	0.85	−0.08 (−0.87, 0.72)
	(0.59, 0.89]	−0.55	0.08	−0.55 (−1.16, 0.06)
	(0.89, 22.92]	−1.07	0.003	−1.07 (−1.75, −0.39)
Sfa 14.0	[−2.36, 1.42]	ref	ref	ref
	(1.42, 1.99]	−0.52	0.15	−0.52 (−1.24, 0.20)
	(1.99, 2.65]	−1.3	<0.001	−1.3 (−1.91, −0.68)
	(2.65, 12.57]	−2.39	<0.001	−2.39 (−3.11, −1.67)
Sfa 16.0	[−8.32, 11.83]	ref	ref	ref
	(11.83, 13.88]	−1.05	0.003	−1.05 (−1.72, −0.37)
	(13.88, 16.02]	−2.42	<0.001	−2.42 (−3.15, −1.69)
	(16.02, 41.30]	−3.2	<0.001	−3.2 (−3.86, −2.54)
Sfa 18.0	[−4.20, 5.27]	ref	ref	ref
	(5.27, 6.35]	−1.97	<0.001	−1.97 (−2.61, −1.33)
	(6.35, 7.50]	−3.28	<0.001	−3.28 (−3.92, −2.64)
	(7.50, 19.08]	−4.43	<0.001	−4.43 (−5.05, −3.81)

Model Adjusted for sex, age, ethnic/race, marital, family income-to-poverty ratio, education levels, BMI, alcohol consumption status, and smoking consumption status.

In the weighted univariate logistic regression, after adjusting for potential confounding factors, SFAs and OSA were significantly correlated. The estimate was −0.021, the *p* < 0.001, and the OR (95% CI) was 0.98 (0.97, 0.99). As shown in Table 4.

**Table 4:** Multivariate logistic regression results of LC9 and OSA.

Parameter	Estimate	*P-*value	OR (95% CI)
LC9	−0.021	<0.001	0.98 (0.97, 0.99)

Model Adjusted for sex, age, ethnic/race, marital, family income-to-poverty ratio, education levels, BMI, alcohol consumption status, and smoking consumption status.

### 3.3 Multivariate logistic regression results of SFAs and OSA

In the weighted multivariable logistic regression, after adjusting for potential confounding factors, SFAs were significantly associated with OSA. We report the quantile levels at which SFAs and OSA were statistically associated, along with the *p*-values and OR (95% CI). Compared with Q1, Total sfat [Model 1, Q3, 0.03, 1.49 (1.03, 2.15); Model 2, Q3, 0.04, 1.47 (1.01, 2.13)], Sfa 12.0 [Model 1, Q2, 0.02, 0.81 (0.68, 0.97), Q3, 0.004, 0.72 (0.58, 0.89), Q4 0.03, 0.75 (0.58, 0.97); Model 2, Q3, 0.02, 0.77 (0.62, 0.96); Model 3, Q4, 0.04, 0.77 (0.60, 0.98)], Sfa 14.0 [Model 1, Q4, 0.04, 0.71 (0.52, 0.99); Model 2, Q4, 0.03, 0.69 (0.50, 0.96); Model 3, Q4, 0.03, 0.66 (0.46, 0.95)], Sfa 16.0 [Model 1, Q2, 0.01, 0.80 (0.67, 0.95), Q3, 0.02, 0.74 (0.59, 0.94); Model 2, Q2, 0.03, 0.82 (0.69, 0.98), Q3, 0.02, 0.75 (0.59, 0.95); Model 3, Q2, 0.02, 0.81 (0.68, 0.97), Q3, 0.05, 0.77 (0.59, 1.00)]. As shown in Table 5.

**Table 5:** Multivariate logistic regression results of saturated fatty acids and OSA.

Parameter		Model 1		Model 2		Model 3	
		*P*-value	OR (95% CI)	*P*-value	OR (95% CI)	*P*-value	OR (95% CI)
Total sfat	[−17.16, 21.15]	ref	ref	ref	ref	ref	ref
	(21.15, 25.31]	0.56	1.07 (0.84, 1.37)	0.72	1.04 (0.81, 1.34)	0.59	1.07 (0.82, 1.39)
	(25.31, 29.66]	0.03	1.49 (1.03, 2.15)	0.04	1.47 (1.01, 2.13)	0.12	1.37 (0.90, 2.08)
	(29.66, 75.86]	0.2	1.38 (0.84, 2.26)	0.21	1.37 (0.83, 2.26)	0.43	1.24 (0.70, 2.19)
Sfa 4.0	[−0.92, 0.28]	ref	ref	ref	ref	ref	ref
	(0.28, 0.45]	0.42	0.92 (0.75, 1.13)	0.75	0.97 (0.79, 1.19)	0.76	0.97 (0.78, 1.21)
	(0.45, 0.66]	0.34	0.89 (0.69, 1.14)	0.56	0.93 (0.72, 1.20)	0.71	0.95 (0.70, 1.28)
	(0.66, 4.18]	0.44	0.89 (0.66, 1.20)	0.66	0.94 (0.69, 1.27)	0.98	1.00 (0.71, 1.41)
Sfa 6.0	[−0.49, 0.16]	ref	ref	ref	ref	ref	ref
	(0.16, 0.26]	0.48	0.92 (0.72, 1.17)	0.52	0.93 (0.73, 1.18)	0.77	0.97 (0.75, 1.24)
	(0.26, 0.38]	0.71	0.95 (0.71, 1.26)	0.71	0.95 (0.72, 1.26)	0.97	1.00 (0.73, 1.36)
	(0.38, 2.45]	0.69	1.07 (0.75, 1.54)	0.75	1.06 (0.74, 1.50)	0.61	1.10 (0.75, 1.61)
Sfa 8.0	[−0.35, 0.14]	ref	ref	ref	ref	ref	ref
	(0.14, 0.21]	0.66	1.06 (0.81, 1.39)	0.68	1.06 (0.80, 1.39)	0.49	1.10 (0.82, 1.49)
	(0.21, 0.31]	0.69	0.93 (0.65, 1.33)	0.75	0.95 (0.66, 1.35)	0.85	1.03 (0.71, 1.52)
	(0.31, 3.62]	0.55	0.87 (0.55, 1.38)	0.67	0.91 (0.58, 1.43)	0.82	0.95 (0.58, 1.55)
Sfa 10.0	[−0.65, 0.27]	ref	ref	ref	ref	ref	ref
	(0.27, 0.41]	0.26	1.17 (0.88, 1.56)	0.25	1.18 (0.89, 1.56)	0.44	1.12 (0.82, 1.54)
	(0.41, 0.59]	0.19	1.30 (0.88, 1.92)	0.21	1.28 (0.87, 1.91)	0.29	1.24 (0.81, 1.90)
	(0.59, 3.28]	0.15	1.41 (0.88, 2.24)	0.18	1.38 (0.86, 2.23)	0.2	1.38 (0.82, 2.31)
Sfa 12.0	[−1.03, 0.37]	ref	ref	ref	ref	ref	ref
	(0.37, 0.59]	0.02	0.81 (0.68, 0.97)	0.07	0.85 (0.71, 1.01)	0.09	0.85 (0.70, 1.03)
	(0.59, 0.89]	0.004	0.72 (0.58, 0.89)	0.02	0.77 (0.62, 0.96)	0.04	0.77 (0.60, 0.98)
	(0.89, 22.92]	0.03	0.75 (0.58, 0.97)	0.08	0.80 (0.61, 1.03)	0.11	0.81 (0.61, 1.06)
Sfa 14.0	[−2.36, 1.42]	ref	ref	ref	ref	ref	ref
	(1.42, 1.99]	0.38	0.91 (0.73, 1.13)	0.33	0.90 (0.72, 1.12)	0.24	0.86 (0.67, 1.12)
	(1.99, 2.65]	0.31	0.86 (0.64, 1.16)	0.29	0.85 (0.63, 1.15)	0.16	0.80 (0.58, 1.11)
	(2.65, 12.57]	0.04	0.71 (0.52, 0.99)	0.03	0.69 (0.50, 0.96)	0.03	0.66 (0.46, 0.95)
Sfa 16.0	[−8.32, 11.83]	ref	ref	ref	ref	ref	ref
	(11.83, 13.88]	0.01	0.80 (0.67, 0.95)	0.03	0.82 (0.69, 0.98)	0.02	0.81 (0.68, 0.97)
	(13.88, 16.02]	0.02	0.74 (0.59, 0.94)	0.02	0.75 (0.59, 0.95)	0.05	0.77 (0.59, 1.00)
	(16.02, 41.30]	0.45	0.89 (0.65, 1.21)	0.48	0.90 (0.66, 1.23)	0.45	0.88 (0.61, 1.26)
Sfa 18.0	[−4.20, 5.27]	ref	ref	ref	ref	ref	ref
	(5.27, 6.35]	0.6	1.04 (0.88, 1.23)	0.34	1.09 (0.91, 1.30)	0.63	0.96 (0.80, 1.15)
	(6.35, 7.50]	0.46	1.07 (0.89, 1.28)	0.35	1.09 (0.90, 1.32)	0.49	0.93 (0.75, 1.16)
	(7.50, 19.08]	0.45	1.10 (0.85, 1.43)	0.58	1.08 (0.82, 1.42)	0.34	0.87 (0.64, 1.18)

Model 1, No adjustment for any potential influence factors. Model 2, Adjusted for Sex, Age and Ethnic/race. Model Adjusted for Sex, Age, Ethnic/race, Marital, Family income-to-poverty ratio, Education levels, LC9, BMI, alcohol consumption status, and smoking consumption status.

### 3.4 RCS analysis

**Figure 2:**
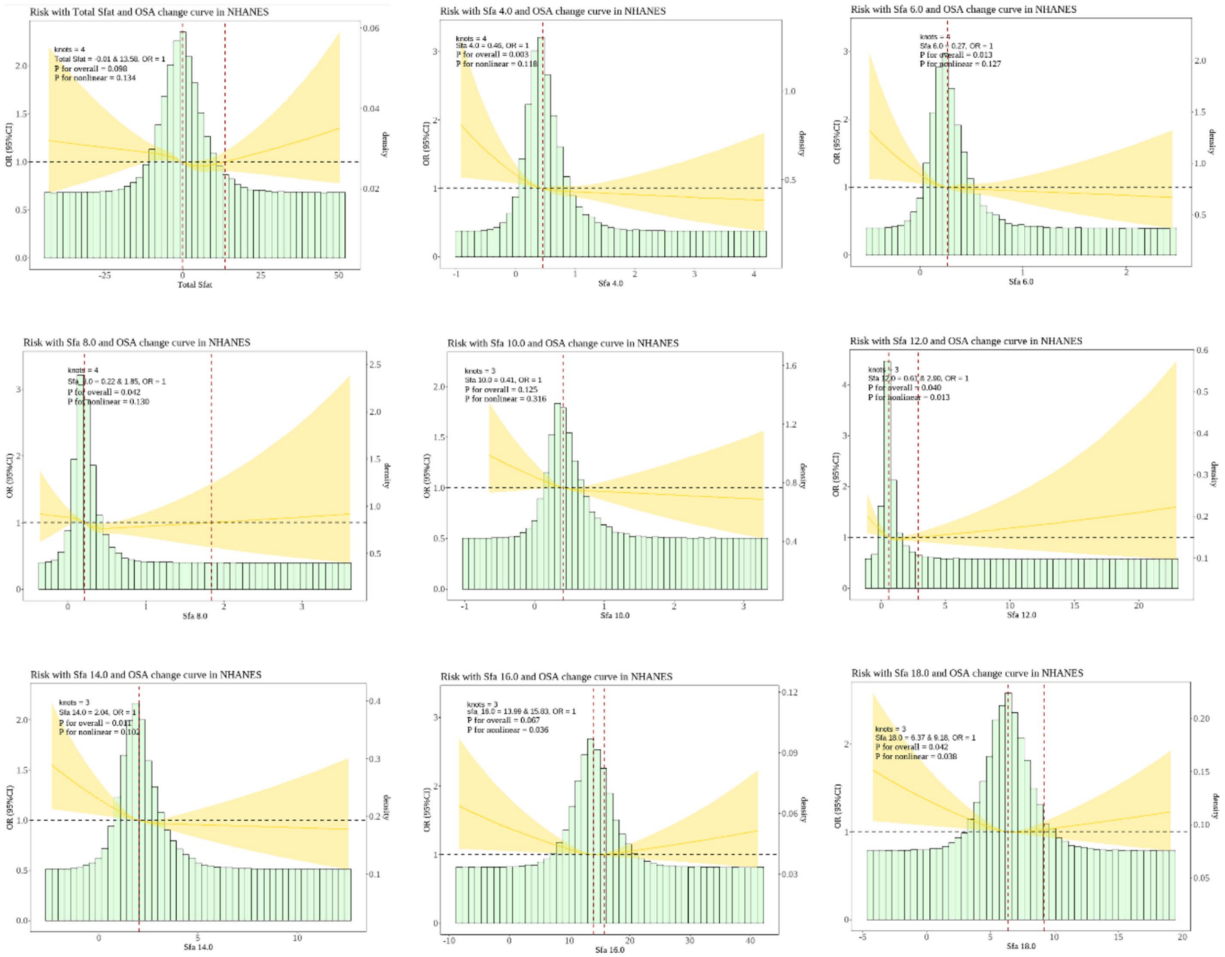
Dose–response relationships between dietary saturated fatty acids and their subtypes and Obstructive sleep apnea (OSA). OR (95% CI) (shaded areas) were adjusted for Sex, Age, Ethnic/race, Marital, Family income-to-poverty ratio, Education levels, BMI, alcohol consumption status, LC9 and smoking consumption status. Ref indicate the minimal threshold for the beneficial association with estimated OR = 1.0 OR, odds ratio.

The RCS analysis disclosed that Sfa 12.0, Sfa 16.0, and Sfa 18.0 exhibited a nonlinear relationship with OSA (P-Nonlinear = 0.013). The dietary intake of Sfa 12.0, Sfa 16.0, and Sfa 18.0 was associated with a “U”-shaped risk of OSA. As shown in Figure 2.

### 3.5 Multivariate logistic regression in dietary SFAs with OSA in different subgroups

Subgroup analyses revealed that no significant interaction was found between SFAs and OSA. Among participants aged 45–64, Total sfat [Q3, 0.05, 2.02 (1.01, 4.11)]; Sfa 14.0 [Q2, 0.05, 0.67 (0.45, 1.00), Q3, 0.05, 0.62 (0.38, 1.00), Q4, 0.01, 0.48 (0.27, 0.84)); Sfa 16.0 (Q3, 0.02, 0.55 (0.33, 0.91), Q4, 0.04, 0.56 (0.33, 0.96)]. Among those aged ≥65, Sfa 6.0 [Q2, 0.02, 0.53 (0.31, 0.91), Q3, 0.03, 0.44 (0.21, 0.92), Q4, 0.03, 0.42 (0.20, 0.91)]; Sfa 18.0 [Q4, 0.04, 0.57 (0.33, 0.98)]. As presented in Table 6. Among female participants, Sfa 12.0 [Q3, 0.04, 0.66 (0.45, 0.99)]. As shown in Table 7. Among white people participants, Sfa 12.0 [Q3, 0.02, 0.66 (0.48, 0.91)], Sfa 14.0 [Q4, 0.01, 0.53 (0.34, 0.84)], Sfa 16.0 [Q2, 0.05, 0.78 (0.61, 1.00); Q3, 0.02, 0.66 (0.47, 0.94)]. Among Mexican people participants, Sfa 4.0 [Q2, 0.04, 0.53 (0.29, 0.95)]. Among Black people participants, Total sfat [Q4, 0.04, 2.55 (1.07, 6.11)], Sfa 18.0 [Q3, 0.04, 0.74 (0.56, 0.99)]. As shown in Table 8.

**Table 6:** Multifactorial logistic regression of age differences in dietary saturated fatty acids and their subtype (Age) with OSA.

Parameter		Q1		Q2		Q3		Q4	P for interaction
			*P*-value	OR (95% CI)	*P*-value	OR (95% CI)	*P*-value	OR (95% CI)	
Total sfat		[−17.16, 21.15]		(21.15, 25.31]		(25.31, 29.66]		(29.66, 75.86]	0.73
	20–44	ref	0.48	0.88 (0.61, 1.28)	0.53	1.18 (0.68, 2.06)	0.53	0.78 (0.35, 1.77)	
	45–64	ref	0.15	1.38 (0.88, 2.15)	0.05	2.02 (1.01, 4.11)	0.07	2.22 (0.92, 5.38)	
	≥ 65	ref	1	1.00 (0.55, 1.83)	0.91	0.95 (0.39, 2.35)	0.84	1.11 (0.37, 3.34)	
Sfa 4.0		[−0.92, 0.28]		(0.28, 0.45]		(0.45, 0.66]		(0.66, 4.18]	0.62
	20–44	ref	0.07	0.74 (0.53, 1.02)	0.24	0.79 (0.52, 1.20)	0.3	0.79 (0.50, 1.26)	
	45–64	ref	0.51	1.13 (0.77, 1.65)	0.75	1.08 (0.66, 1.76)	0.57	1.13 (0.71, 1.80)	
	≥ 65	ref	0.14	1.43 (0.87, 2.33)	0.25	1.46 (0.74, 2.88)	0.13	1.86 (0.82, 4.20)	
Sfa 6.0		[−0.49, 0.16]		(0.16, 0.26]		(0.26, 0.38]		(0.38, 2.45]	0.84
	20–44	ref	0.74	1.07 (0.71, 1.60)	0.58	0.58	0.65	1.13 (0.64, 2.00)	
	45–64	ref	0.9	1.03 (0.67, 1.57)	0.76	1.07 (0.67, 1.73)	0.3	1.33 (0.76, 2.33)	
	≥ 65	ref	0.02	0.53 (0.31, 0.91)	0.03	0.44 (0.21, 0.92)	0.03	0.42 (0.20, 0.91)	
Sfa 8.0		[−0.35, 0.14]		(0.14, 0.21]		(0.21, 0.31]		(0.31, 3.62]	0.68
	20–44	ref	0.83	0.96 (0.62, 1.46)	0.56	0.87 (0.53, 1.44)	0.73	0.91 (0.50, 1.65)	
	45–64	ref	0.35	1.23 (0.78, 1.93)	0.75	1.09 (0.63, 1.90)	0.84	0.94 (0.49, 1.80)	
	≥ 65	ref	0.25	1.44 (0.75, 2.77)	0.21	1.77 (0.70, 4.45)	0.53	1.38 (0.48, 3.98)	
Sfa 10.0		[−0.65, 0.27]		(0.27, 0.41]		(0.41, 0.59]		(0.59, 3.28]	0.41
	20–44	ref	0.36	1.23 (0.77, 1.94)	0.21	1.46 (0.79, 2.70)	0.26	1.48 (0.72, 3.07)	
	45–64	ref	0.58	0.87 (0.52, 1.46)	0.8	0.93 (0.51, 1.70)	0.81	0.92 (0.46, 1.87)	
	≥ 65	ref	0.13	1.66 (0.84, 3.26)	0.21	1.71 (0.71, 4.14)	0.06	2.76 (0.94, 8.11)	
Sfa 12.0		[−1.03, 0.37]		(0.37, 0.59]		(0.59, 0.89]		(0.89, 22.92]	0.18
	20–44	ref	0.16	0.78 (0.54, 1.11)	0.07	0.71 (0.49, 1.03)	0.09	0.70 (0.47, 1.06)	
	45–64	ref	0.58	0.92 (0.66, 1.28)	0.95	0.99 (0.67, 1.46)	0.77	0.94 (0.62, 1.44)	
	≥ 65	ref	0.87	0.95 (0.53, 1.73)	0.11	0.53 (0.24, 1.18)	0.57	0.78 (0.31, 1.94)	
Sfa 14.0		[−2.36, 1.42]		(1.42, 1.99]		(1.99, 2.65]		(2.65, 12.57]	0.89
	20–44	ref	0.4	1.16 (0.80, 1.67)	0.81	1.05 (0.66, 1.68)	0.92	0.98 (0.58, 1.65)	
	45–64	ref	0.05	0.67 (0.45, 1.00)	0.05	0.62 (0.38, 1.00)	0.01	0.48 (0.27, 0.84)	
	≥ 65	ref	0.09	0.66 (0.40, 1.07)	0.15	0.63 (0.33, 1.21)	0.06	0.45 (0.20, 1.04)	
Sfa 16.0		[−8.32, 11.83]		(11.83, 13.88]		(13.88, 16.02]		(16.02, 41.30]	0.29
	20–44	ref	0.42	0.87 (0.62, 1.24)	0.15	0.73 (0.47, 1.14)	0.72	1.11 (0.59, 2.09)	
	45–64	ref	0.08	0.70 (0.47, 1.04)	0.02	0.55 (0.33, 0.91)	0.04	0.56 (0.33, 0.96)	
	≥ 65	ref	0.77	0.93 (0.57, 1.53)	0.19	1.60 (0.76, 3.34)	0.36	1.44 (0.63, 3.28)	
Sfa 18.0		[−4.20, 5.27]		(5.27, 6.35]		(6.35, 7.50]		(7.50, 19.08]	0.27
	20–44	ref	0.26	0.85 (0.64, 1.14)	0.47	0.89 (0.64, 1.24)	0.73	0.93 (0.62, 1.42)	
	45–64	ref	0.34	1.19 (0.82, 1.71)	0.46	1.14 (0.79, 1.63)	0.99	1.00 (0.60, 1.66)	
	≥ 65	ref	0.22	0.78 (0.52, 1.18)	0.07	0.66 (0.41, 1.05)	0.04	0.57 (0.33, 0.98)	

Model Adjusted for Sex, LC9, Ethnic/race, Marital, Family income-to-poverty ratio, Education levels, BMI, alcohol consumption status, and smoking consumption status.

**Table 7:** Multifactorial logistic regression of age differences in dietary saturated fatty acids and their subtype (sex) with OSA.

Parameter		Q1	Q2		Q3		Q4		P for interaction
			*P*-value	OR (95% CI)	*P*-value	OR (95% CI)	*P*-value	OR (95% CI)	
Total sfat		[−17.16, 21.15]		(21.15, 25.31]		(25.31, 29.66]		(29.66, 75.86]	0.89
	Female	ref	0.56	1.15 (0.70, 1.88)	0.07	1.76 (0.94, 3.30)	0.07	2.07 (0.94, 4.55)	
	Male	ref	0.99	1.00 (0.67, 1.48)	0.84	1.05 (0.61, 1.82)	0.44	0.75 (0.34, 1.66)	
Sfa 4.0		[−0.92, 0.28]		(0.28, 0.45]		(0.45, 0.66]		(0.66, 4.18]	0.44
	Female	ref	0.57	0.91 (0.65, 1.29)	0.35	0.82 (0.54, 1.26)	0.23	0.73 (0.43, 1.25)	
	Male	ref	0.95	0.99 (0.76, 1.30)	0.85	1.04 (0.70, 1.54)	0.29	1.26 (0.80, 1.99)	
Sfa 6.0		[−0.49, 0.16]		(0.16, 0.26]		(0.26, 0.38]		(0.38, 2.45]	0.62
	Female	ref	0.96	0.99 (0.68, 1.45)	0.76	0.94 (0.59, 1.48)	0.48	1.20 (0.70, 2.06)	
	Male	ref	0.82	0.97 (0.70, 1.33)	0.71	1.07 (0.73, 1.57)	0.78	1.07 (0.65, 1.77)	
Sfa 8.0		[−0.35, 0.14]		(0.14, 0.21]		(0.21, 0.31]		(0.31, 3.62]	0.66
	Female	ref	0.73	1.07 (0.69, 1.68)	0.86	1.04 (0.61, 1.78)	0.49	0.80 (0.40, 1.58)	
	Male	ref	0.65	1.08 (0.76, 1.54)	0.96	1.01 (0.62, 1.65)	0.79	1.08 (0.59, 1.98)	
Sfa 10.0		[−0.65, 0.27]		(0.27, 0.41]		(0.41, 0.59]		(0.59, 3.28]	0.74
	Female	ref	0.77	1.07 (0.65, 1.75)	0.43	1.29 (0.66, 2.54)	0.18	1.62 (0.78, 3.39)	
	Male	ref	0.42	1.16 (0.79, 1.70)	0.45	1.20 (0.73, 1.97)	0.67	1.15 (0.57, 2.35)	
Sfa 12.0		[−1.03, 0.37]		(0.37, 0.59]		(0.59, 0.89]		(0.89, 22.92]	0.86
	Female	ref	0.13	0.77 (0.55, 1.10)	0.04	0.66 (0.45, 0.99)	0.11	0.71 (0.46, 1.10)	
	Male	ref	0.64	0.94 (0.71, 1.24)	0.42	0.89 (0.65, 1.21)	0.55	0.90 (0.63, 1.29)	
Sfa 14.0		[−2.36, 1.42]		(1.42, 1.99]		(1.99, 2.65]		(2.65, 12.57]	0.93
	Female	ref	0.76	0.94 (0.61, 1.45)	0.57	0.87 (0.51, 1.47)	0.15	0.65 (0.36, 1.19)	
	Male	ref	0.21	0.82 (0.60, 1.13)	0.17	0.76 (0.50, 1.14)	0.16	0.68 (0.39, 1.19)	
Sfa 16.0		[−8.32, 11.83]		(11.83, 13.88]		(13.88, 16.02]		(16.02, 41.30]	0.65
	Female	ref	0.39	0.85 (0.58, 1.26)	0.25	0.79 (0.52, 1.20)	0.35	0.79 (0.48, 1.32)	
	Male	ref	0.08	0.79 (0.60, 1.04)	0.14	0.76 (0.53, 1.10)	0.95	1.01 (0.59, 1.74)	
Sfa 18.0		[−4.20, 5.27]		(5.27, 6.35]		(6.35, 7.50]		(7.50, 19.08]	0.99
	Female	ref	0.66	0.94 (0.68, 1.29)	0.36	0.83 (0.54, 1.28)	0.19	0.72 (0.44, 1.20)	
	Male	ref	0.91	0.99 (0.77, 1.27)	0.68	1.06 (0.78, 1.44)	0.71	1.07 (0.72, 1.59)	

Model Adjusted for Age, LC9, Ethnic/race, Marital, Family income-to-poverty ratio, Education levels, BMI, alcohol consumption status, and smoking consumption status.

**Table 8:** Multifactorial logistic regression of age differences in dietary saturated fatty acids and their subtype (ethnic/race) with OSA.

Parameter		Q1		Q2		Q3		Q4	P for interaction
			*P*-value	OR (95% CI)	*P*-value	OR (95% CI)	*P*-value	OR (95% CI)	
Total sfat		[−17.16, 21.15]		(21.15, 25.31]		(25.31, 29.66]		(29.66, 75.86]	0.16
	White people	ref	0.56	1.09 (0.80, 1.48)	0.08	1.61 (0.93, 2.77)	0.35	1.37 (0.68, 2.77)	
	Black people	ref	0.17	1.48 (0.82, 2.69)	0.07	1.88 (0.95, 3.72)	0.04	2.55 (1.07, 6.11)	
	Mexican people	ref	0.93	0.98 (0.46, 2.08)	0.13	0.53 (0.21, 1.31)	0.11	0.40 (0.12, 1.32)	
	Other	ref	0.22	0.71 (0.40, 1.26)	0.31	0.70 (0.34, 1.45)	0.61	0.78 (0.27, 2.21)	
Sfa 4.0		[−0.92, 0.28]		(0.28, 0.45]		(0.45, 0.66]		(0.66, 4.18]	0.66
	White people	ref	0.85	1.03 (0.77, 1.37)	0.73	1.07 (0.72, 1.60)	0.44	1.18 (0.75, 1.85)	
	Black people	ref	0.77	1.05 (0.73, 1.52)	0.55	0.89 (0.58, 1.35)	0.38	0.76 (0.39, 1.47)	
	Mexican people	ref	0.04	0.53 (0.29, 0.95)	0.09	0.51 (0.23, 1.16)	0.09	0.47 (0.19, 1.20)	
	Other	ref	0.5	0.86 (0.54, 1.36)	0.29	0.74 (0.42, 1.32)	0.44	0.75 (0.34, 1.65)	
Sfa 6.0		[−0.49, 0.16]		(0.16, 0.26]		(0.26, 0.38]		(0.38, 2.45]	0.84
	White people	ref	0.95	0.99 (0.72, 1.36)	0.93	0.98 (0.66, 1.47)	0.81	1.05 (0.65, 1.71)	
	Black people	ref	0.87	1.03 (0.66, 1.63)	0.55	1.16 (0.68, 1.99)	0.7	1.12 (0.60, 2.09)	
	Mexican people	ref	0.87	0.95 (0.41, 2.17)	0.45	0.77 (0.35, 1.72)	0.67	0.84 (0.30, 2.30)	
	Other	ref	0.96	0.99 (0.53, 1.84)	0.57	1.28 (0.51, 3.25)	0.13	2.22 (0.76, 6.53)	
Sfa 8.0		[−0.35, 0.14]		(0.14, 0.21]		(0.21, 0.31]		(0.31, 3.62]	0.59
	White people	ref	0.76	1.05 (0.73, 1.52)	0.57	1.14 (0.71, 1.83)	0.97	1.01 (0.54, 1.88)	
	Black people	ref	0.83	1.05 (0.63, 1.77)	0.38	0.78 (0.44, 1.40)	0.27	0.70 (0.36, 1.38)	
	Mexican people	ref	0.19	1.37 (0.80, 2.37)	0.94	1.02 (0.50, 2.09)	0.87	1.07 (0.42, 2.70)	
	Other	ref	0.79	1.10 (0.53, 2.28)	0.43	0.72 (0.30, 1.71)	0.61	0.78 (0.29, 2.14)	
Sfa 10.0		[−0.65, 0.27]		(0.27, 0.41]		(0.41, 0.59]		(0.59, 3.28]	0.48
	White people	ref	0.38	1.19 (0.79, 1.78)	0.41	1.23 (0.73, 2.06)	0.19	1.51 (0.80, 2.86)	
	Black people	ref	0.58	0.88 (0.53, 1.45)	0.42	1.27 (0.68, 2.38)	0.51	1.29 (0.57, 2.90)	
	Mexican people	ref	0.79	1.09 (0.50, 2.35)	0.87	1.07 (0.39, 2.93)	0.64	1.20 (0.47, 3.06)	
	Other	ref	0.95	0.98 (0.58, 1.67)	0.46	1.32 (0.60, 2.91)	0.41	0.66 (0.22, 1.92)	
Sfa 12.0		[−1.03, 0.37]		(0.37, 0.59]		(0.59, 0.89]		(0.89, 22.92]	0.17
	White people	ref	0.08	0.80 (0.63, 1.03)	0.02	0.66 (0.48, 0.91)	0.1	0.74 (0.50, 1.07)	
	Black people	ref	0.83	0.96 (0.61, 1.50)	0.81	0.95 (0.58, 1.54)	0.72	1.09 (0.64, 1.86)	
	Mexican people	ref	0.27	1.23 (0.81, 1.87)	0.19	1.34 (0.82, 2.19)	0.49	1.21 (0.63, 2.36)	
	Other	ref	0.95	0.99 (0.65, 1.50)	0.46	1.19 (0.73, 1.93)	0.95	0.98 (0.51, 1.87)	
Sfa 14.0		[−2.36, 1.42]		(1.42, 1.99]		(1.99, 2.65]		(2.65, 12.57]	0.36
	White people	ref	0.16	0.80 (0.57, 1.11)	0.14	0.72 (0.46, 1.13)	0.01	0.53 (0.34, 0.84)	
	Black people	ref	0.8	0.94 (0.54, 1.62)	0.74	0.90 (0.47, 1.74)	0.7	0.84 (0.31, 2.25)	
	Mexican people	ref	0.95	1.02 (0.52, 2.01)	0.16	1.67 (0.74, 3.76)	0.17	1.78 (0.72, 4.41)	
	Other	ref	0.93	1.02 (0.65, 1.58)	0.12	0.64 (0.35, 1.15)	0.73	0.84 (0.30, 2.38)	
Sfa 16.0		[−8.32, 11.83]		(11.83, 13.88]		(13.88, 16.02]		(16.02, 41.30]	0.43
	White people	ref	0.05	0.78 (0.61, 1.00)	0.02	0.66 (0.47, 0.94)	0.33	0.80 (0.51, 1.27)	
	Black people	ref	0.29	0.83 (0.57, 1.20)	0.6	0.88 (0.54, 1.45)	0.31	0.78 (0.47, 1.31)	
	Mexican people	ref	0.73	0.91 (0.48, 1.74)	0.54	1.23 (0.54, 2.83)	0.13	1.95 (0.76, 5.03)	
	Other	ref	0.58	0.88 (0.55, 1.42)	0.39	1.26 (0.72, 2.21)	0.95	0.98 (0.44, 2.19)	
Sfa 18.0		[−4.20, 5.27]		(5.27, 6.35]		(6.35, 7.50]		(7.50, 19.08]	0.55
	White people	ref	0.42	0.91 (0.70, 1.17)	0.63	0.94 (0.71, 1.24)	0.4	0.86 (0.58, 1.26)	
	Black people	ref	0.24	0.81 (0.56, 1.17)	0.02	0.55 (0.35, 0.87)	0.1	0.63 (0.35, 1.11)	
	Mexican people	ref	0.78	1.06 (0.63, 1.79)	0.47	1.21 (0.64, 2.30)	0.77	1.09 (0.53, 2.27)	
	Other	ref	0.16	1.41 (0.86, 2.31)	0.48	1.20 (0.70, 2.09)	0.74	1.11 (0.56, 2.18)	

Model adjusted for age, LC9, sex, marital, family income-to-poverty ratio, education levels, BMI, alcohol consumption status, and smoking consumption status.

### 3.6 Mediation analysis

**Figure 3:**
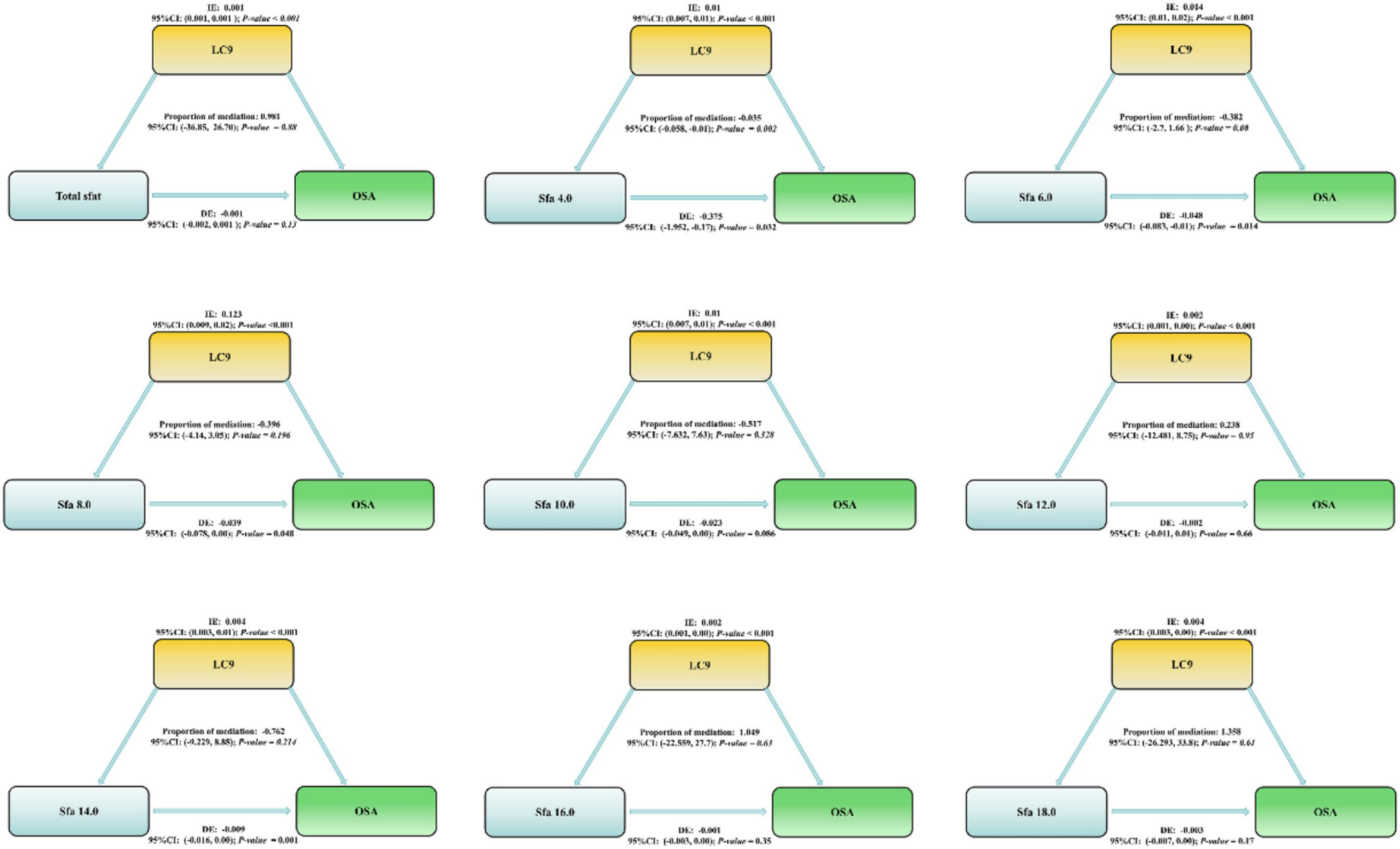
Life’s critical 9 Mediating effects and estimated proportions in the association of dietary intake of saturated fatty acids and their subtypes with OSA. Model Adjusted for Sex, Age, Ethnic/race, Marital, Family income-to-poverty ratio, Education levels, BMI, alcohol consumption status and smoking consumption status. Abbreviations: IE, the estimate of the indirect effect; DE, the estimate of the direct effect; Proportion of mediation = IE/DE + IE, OR, odds ratio.

LC9 exerts a mediating role in the correlation between Sfa4.0 and the risk of OSA. Proportion of mediation: −0.035; 95% CI: (−0.058, −0.01); *p*-value = 0.002. As depicted in Figure 3.

## 4 Discussion

This study explored the association between the intake of saturated fatty acids (SFAs) and obstructive sleep apnea (OSA) among 13,563 adults in NHANES from 2005 to 2008 and 2015 to 2018. We offer two findings based on the general population of the United States. Firstly, total sfat increased the risk of OSA, emphasizing that the dietary intake of Sfa 12.0, Sfa 14.0, and Sfa 16.0 were protective factors for OSA, particularly among participants aged 45–64 and white people. Moreover, Sfa 12.0, Sfa 16.0, and Sfa 18.0 presented a nonlinear relationship with OSA. Secondly, Sfa 12.0 demonstrated superior protective effects in female participants. Additionally, LC9 had a mediating effect on Sfa 4.0 in relation to OSA.

Epidemiological studies have pointed out that dietary intake of SFAs is negatively associated with total sleep duration, resulting in a decrease in the duration of nocturnal slow-wave sleep and an increase in the number of awakenings (35, 36). In a prospective study, an association between SFAs and OSA was demonstrated in stroke patients (37). A higher intake of SFAs can raise the risk of OSA (38), while a lower intake of SFAs can alleviate daytime sleepiness in OSA patients (39). In animal studies, a high-fat diet can influence the expression of clock genes in the central and peripheral nervous systems, disrupting the circadian rhythm (40, 41). The above research results suggest that an increase in dietary fat and SFAs intake has an unfavorable impact on sleep status, manifested as a reduction in sleep time, a decline in sleep quality, and an increase in the risk of OSA. Our research results indicate that dietary intake of SFAs can enhance the risk of OSA.

Our research results indicate that Sfa 12.0, Sfa 14.0, and Sfa 16.0 are protective factors for OSA, particularly among participants aged 45–64 and white people. The most prevalent saturated fatty acids in the American diet are Sfa 12.0, Sfa 14.0, Sfa 16.0, and Sfa 18.0, and their typical food sources have numerous overlaps. For instance, the dietary sources of Sfa 14.0 include palm kernel oil, coconut oil, and butter, while those of Sfa 16.0 include palm kernel oil, milk fat, meat, cocoa butter, soybean oil, and sunflower oil. Sfa 14.0 and Sfa 16.0 have comparable effects on LDL and HDL cholesterol, but have a negligible overall impact on the total cholesterol:HDL cholesterol ratio (42). Compared to other SFAs, Sfa 18.0 can reduce plasma LDL cholesterol levels and has no influence on HDL cholesterol (43, 44). Hence, even though Sfa 18.0 is a type of SFA, it seemingly does not have an adverse effect on CVD risk, possibly because it is partially desaturated to oleate during the metabolic process (43, 45). This study investigated the association between OSA and lipid concentrations in the European Sleep Apnea Database (ESADA) cohort, noting that OSA patients have a higher prevalence of dyslipidemia. The lipid profile disorder mainly manifests as a reduction in HDL-C and varying degrees of elevated LDL-C (46). The severity of OSA is positively correlated with a decrease in serum HDL-C levels (47). Another study mentioned that 5-HT2A receptor antagonists can improve sleep maintenance and sleep efficiency without causing motor coordination disorders. Molecular docking studies discovered that Sfa 14.0 may form favorable interactions with the 5-HT2A receptor and the α1A-adrenergic receptor (48). Sfa 14.0 demonstrates an affinity for the 5-HT2A receptor and can prolong the sleep time of mice in a dose-independent manner (48). This might imply that Sfa 14.0 can influence sleep through this mechanism. To date, we have not found studies that can directly prove that Sfa 12.0 can reduce the risk of OSA. However, we have identified several possible reasons. From an epidemiological perspective, saturated fatty acids are closely related to hypertension (49). Our previous research also indicated that the dietary intake of Sfa 12.0 can lower hypertension in women (25). This might explain the superior protective effect of Sfa 12.0 in female participants. Sleep quality is regulated by blood pressure and heart rate (22). In *in vivo* studies, Sfa 12.0 (1–10 mg/kg, intravenous injection, once) has been reported to reduce blood pressure and heart rate in rats (50). *In vitro* studies have shown that Sfa 12.0 (0.06–125 μg/mL, 24–72 h) exhibits stronger antibacterial activity in *P. acnes*, *S. aureus*, and *S. epidermidis* (51). Although this study did not directly link Sfa 12.0 with sleep disorders, considering that sleep disorders may be related to infection and inflammation, the antibacterial properties of Sfa 12.0 may have an indirect impact on improving the sleep environment (51). Since our research cannot provide basic research evidence, cross-sectional epidemiological studies can only identify the association between Sfa 12.0 and OSA. More basic research is needed to clarify the mechanism of action of Sfa 12.0 on OSA. We speculate that Sfa 12.0 may indirectly affect sleep by influencing these physiological parameters. Our research results show that the mediating effect of LC9 between Sfa 12.0 and OSA is not statistically significant. This might be due to differences in the study population, insufficient sample size, and the lack of long-term follow-up.

Our research results indicate a nonlinear “U”-shaped relationship between the dietary intakes of Sfa 12.0, Sfa 16.0, and Sfa 18.0 and OSA, signifying that the risk of OSA initially decreases and then increases with the increase of the dietary intakes of Sfa 12.0, Sfa 16.0, and Sfa 18.0. The possible reason is that when the dietary intake of SFAs is relatively low, Sfa 12.0, Sfa 16.0, and Sfa 18.0 can exert protective effects on OSA. For instance, Sfa 12.0 has the most significant effect in increasing high-density lipoprotein (HDL) cholesterol and can reduce the ratio of total cholesterol to HDL cholesterol (52). Sfa 14.0 might extend to Sfa 16.0, and they can exert similar functions (37). Another study discovered that glial mitochondrial *β*-oxidation is essential for sleep, and long-chain saturated fatty acids can rescue sleep reduction caused by glial Drp1 RNAi, indicating the important role of reduced lipid accumulation and catabolism in sleep loss (53). This seemingly supports the conclusion that long-chain fatty acids have a protective effect on OSA. Nevertheless, the dietary intake of fatty acids is a complex mixture. The existing knowledge is that SFAs as a whole are detrimental to cardiovascular health and increase the risk of OSA (24). Hence, we speculate that when the dietary intake increases, the healthy subtypes of SFAs cannot counteract the adverse effects of SFAs on lipid metabolism. Moreover, due to the complexity of individual diets, studies based on questionnaires cannot rule out the influence of other dietary factors.

Another significant discovery was that the cardiovascular health score (LC9) exerted a mediating effect in the relationship between Sfa4.0 and OSA. There is scarce direct evidence regarding Sfa4.0 in OSA; however, Sfa4.0 belongs to short-chain saturated fatty acids, and saturated fatty acids of similar lengths share similar physiopathological mechanisms. Short-chain fatty acids are the main by-products of intestinal fiber fermentation and are associated with increased intestinal permeability, metabolic dysregulation, and cardiovascular diseases, which are more prevalent among the elderly and may impact sleep efficiency (16, 54). Research has pointed out that short-chain fatty acids (Sfa4.0) vary throughout the day, with the highest concentration emerging earlier and continuously decreasing throughout the day, which might influence the expression of circadian genes (16). Nevertheless, higher concentrations of Sfa4.0 are correlated with lower sleep efficiency and longer sleep onset latency, suggesting that short-chain fatty acids might affect sleep continuity and contribute to promoting short-chain saturated fatty acids as biomarkers of insomnia (16). Additionally, previous studies have demonstrated that cardiovascular diseases can increase the risk of OSA, which has been verified (19). Due to the insufficient research on Sfa4.0, we are unable to conduct a more detailed discussion. Fortunately, studies on SFAs and cardiovascular diseases are relatively comprehensive, and an increased cardiovascular health risk facilitates the occurrence of OSA. Our current study can only support the notion that short-chain saturated fatty acids can promote the occurrence of OSA by deteriorating cardiovascular health. Furthermore, the protective effects of Sfa 12.0, Sfa 14.0, and Sfa 16.0 were more pronounced among participants aged 45–64 and white people. This might be closely related to the dietary habits of the participants. Both subgroups of participants aged 45–64 and white people tend to have stable social status and cultural differences, and possess better dietary habits and opportunities to maintain good physical health. In a study, the plasma metabolomics characteristics of black and white participants revealed that dietary habits and food preferences might have a significant impact on the types/distribution of lipid metabolites (55). Additionally, studies have found that there are differences in fatty acid-derived desaturation indices among races, and insulin-resistant black South African women exhibit fatty acid patterns that are typically associated with higher insulin sensitivity in the European population (56). In subsequent studies, we will narrow the research scope and explore the racial differences in the effects of SFAs on OSA.

This study boasts several notable strengths. Firstly, it is grounded in a large-scale study of complex samples, offering more dependable results. Such a research design not only enhances the generalizability of the findings but also furnishes the necessary basis for comprehending the influencing factors under different sample characteristics. Moreover, this study specifically focuses on the variance in the carbon chain lengths of saturated fatty acids (SFA), this novel perspective enables us to conduct in-depth analyses of the distinct influences of SFAs with different carbon chain lengths on OSA. Simultaneously, the study has corrected and weighted the nutrient components and demographic data, ensuring the scientificity and accuracy of the outcomes. However, there are limitations to be recognized in this cross-sectional study. The research discovered that there might exist complex internal interaction mechanisms among SFA and its subtypes, a factor that could impact the interpretation of the ultimate results. Additionally, the intake of SFA was measured via self-reported dietary frequency questionnaires (FFQ), which might entail recall bias and thereby influence the accuracy of the data. It is worth noting that observational studies cannot rule out the possibility of reverse causation. Furthermore, in the NHANES survey, there could be Neyman bias, which might lead to misleading results for specific populations. Finally, this study solely relies on two measurements of dietary intake and lacks sufficient follow-up data, thereby restricting an in-depth exploration of the relationship between SFA and OSA. Based on these findings, the intake of SFAs was significantly and positively associated with the risk of OSA, which supports the importance of dietary fat quality for cardiovascular health and OSA and provides a new scientific basis for dietary guidance. Future clinical interventions should take into account patients’ age, gender, and ethnicity to develop individualized dietary intervention plans. Reducing the intake of SFAs may decrease the prevalence of OSA, thereby reducing the economic burden on the health system. Future studies should focus on the mechanism of action of SFAs on the risk of OSA and further evaluate the independent and interactive effects of different subtypes of fatty acids.

## 5 Conclusion

In conclusion, our results indicate that the mixture of SFAs presents as a risk factor for OSA, with a correlation between Total sfat and the increased risk of OSA. We discovered that the subtypes of long chain saturated fatty acids are protective factors for OSA. Additionally, the mediation analysis suggests that the association between Sfa4.0 and the risk of OSA might be mediated by Life’s Crucial 9. These findings identify the risk factors of OSA and suggest that cardiovascular health is a potential mechanism that adversely affects OSA.

## Data Availability

Publicly available datasets were analyzed in this study. This data can be found at: All data entered into the analysis were from NHANES, which is publicly accessible to: https://www.cdc.gov/nchs/nhanes/index.htm.

## References

[1] Benjafield AV Ayas NT Eastwood PR Heinzer R Ip M Morrell MJ. Estimation of the global prevalence and burden of obstructive sleep apnoea: a literature-based analysis. Lancet Respir Med. (2019) 7:687–98. doi: 10.1016/S2213-2600(19)30198-5, PMID: 31300334 PMC7007763

[2] Barros D Garcia-Rio F. Obstructive sleep apnea and dyslipidemia: from animal models to clinical evidence. Sleep. (2019) 42:zsy236. doi: 10.1093/sleep/zsy236, PMID: 30476296

[3] Gottlieb DJ Punjabi NM. Diagnosis and Management of Obstructive Sleep Apnea: a review. JAMA. (2020) 323:1389–400. doi: 10.1001/jama.2020.3514, PMID: 32286648

[4] Javaheri S Barbe F Campos-Rodriguez F Dempsey JA Khayat R Javaheri S. Sleep apnea: types, mechanisms, and clinical cardiovascular consequences. J Am Coll Cardiol. (2017) 69:841–58. doi: 10.1016/j.jacc.2016.11.069, PMID: 28209226 PMC5393905

[5] Logan AG Perlikowski SM Mente A Tisler A Tkacova R Niroumand M. High prevalence of unrecognized sleep apnoea in drug-resistant hypertension. J Hypertens. (2001) 19:2271–7. doi: 10.1097/00004872-200112000-00022, PMID: 11725173

[6] Bonsignore MR. Obesity and obstructive sleep apnea. Handb Exp Pharmacol. (2022) 274:181–201. doi: 10.1007/164_2021_558, PMID: 34697666

[7] Rasaei N Samadi M Khadem A Badrooj N Hassan ZM Ghaffarian-Ensaf R. The association between cholesterol/saturated fat index (CSI) and quality of sleep, and circadian rhythm among overweight and obese women: a cross-sectional study. J Health Popul Nutr. (2023) 42:75. doi: 10.1186/s41043-023-00414-1, PMID: 37501196 PMC10375646

[8] Arvanitis M Lowenstein CJ. Dyslipidemia. Ann Intern Med. (2023) 176:ITC81–96. doi: 10.7326/AITC202306200, PMID: 37307585

[9] Zhao Y Yang YY Yang BL Du YW Ren DW Zhou HM. Efficacy and safety of berberine for dyslipidemia: study protocol for a randomized double-blind placebo-controlled trial. Trials. (2021) 22:85. doi: 10.1186/s13063-021-05028-8, PMID: 33482853 PMC7825207

[10] Zhang X Wang S Xu H Yi H Guan J Yin S. Metabolomics and microbiome profiling as biomarkers in obstructive sleep apnoea: a comprehensive review. Eur Respir Rev. (2021) 30:200220. doi: 10.1183/16000617.0220-2020, PMID: 33980666 PMC9489097

[11] Frank S Gonzalez K Lee-Ang L Young MC Tamez M Mattei J. Diet and sleep physiology: public health and clinical implications. Front Neurol. (2017) 8:393. doi: 10.3389/fneur.2017.00393, PMID: 28848491 PMC5554513

[12] Dhar P Tayade AB Kumar J Chaurasia OP Srivastava RB Singh SB. Nutritional profile of phytococktail from trans-Himalayan plants. PLoS One. (2013) 8:e83008. doi: 10.1371/journal.pone.0083008, PMID: 24376624 PMC3871620

[13] Condo D Lastella M Aisbett B Stevens A Roberts S. Sleep duration and quality are associated with nutrient intake in elite female athletes. J Sci Med Sport. (2022) 25:345–50. doi: 10.1016/j.jsams.2021.11.045, PMID: 34952801

[14] Bove C Jain V Younes N Hynes M. What you eat could affect your sleep: dietary findings in patients with newly diagnosed obstructive sleep apnea. Am J Lifestyle Med. (2021) 15:305–12. doi: 10.1177/1559827618765097, PMID: 34025323 PMC8120616

[15] Jenkins JB Omori T Guan Z Vgontzas AN Bixler EO Fang J. Sleep is increased in mice with obesity induced by high-fat food. Physiol Behav. (2006) 87:255–62. doi: 10.1016/j.physbeh.2005.10.010, PMID: 16360185

[16] Magzal F Even C Haimov I Agmon M Asraf K Shochat T. Associations between fecal short-chain fatty acids and sleep continuity in older adults with insomnia symptoms. Sci Rep. (2021) 11:4052. doi: 10.1038/s41598-021-83389-5, PMID: 33603001 PMC7893161

[17] Cummings JH Macfarlane GT. The control and consequences of bacterial fermentation in the human colon. J Appl Bacteriol. (1991) 70:443–59. doi: 10.1111/j.1365-2672.1991.tb02739.x, PMID: 1938669

[18] Huang T Mariani S Redline S. Sleep irregularity and risk of cardiovascular events: the multi-ethnic study of atherosclerosis. J Am Coll Cardiol. (2020) 75:991–9. doi: 10.1016/j.jacc.2019.12.054, PMID: 32138974 PMC7237955

[19] Peker Y Akdeniz B Altay S Balcan B Basaran O Baysal E. Obstructive sleep apnea and cardiovascular disease: where do we stand? Anatol J Cardiol. (2023) 27:375–89. doi: 10.14744/AnatolJCardiol.2023.3307, PMID: 37284828 PMC10339137

[20] Lloyd-Jones DM Allen NB Anderson C Black T Brewer LC Foraker RE. Life's essential 8: updating and enhancing the American Heart Association's construct of cardiovascular health: a presidential advisory from the American Heart Association. Circulation. (2022) 146:e18–43. doi: 10.1161/CIR.0000000000001078, PMID: 35766027 PMC10503546

[21] Ge J Peng W Lu J. Predictive value of Life's crucial 9 for cardiovascular and all-cause mortality: a prospective cohort study from the NHANES 2007 to 2018. J Am Heart Assoc. (2024) 13:e036669. doi: 10.1161/JAHA.124.036669, PMID: 39377201 PMC11935597

[22] Wang S Niu X Zhang P Su D Zhang J Liu W. Analysis of OSAS incidence and influential factors in middle-aged and elderly patients with hypertension. Minerva Med. (2019) 110:115–20. doi: 10.23736/S0026-4806.18.05635-5, PMID: 29696938

[23] Eckel RH Jakicic JM Ard JD de Jesus JM Houston MN Hubbard VS. 2013 AHA/ACC guideline on lifestyle management to reduce cardiovascular risk: a report of the American College of Cardiology/American Heart Association task force on practice guidelines. Circulation. (2014) 129:S76–99. doi: 10.1161/01.cir.0000437740.48606.d1, PMID: 24222015

[24] Briggs MA Petersen KS Kris-Etherton PM. Saturated fatty acids and cardiovascular disease: replacements for saturated fat to reduce cardiovascular risk. Healthcare. (2017) 5:29. doi: 10.3390/healthcare5020029, PMID: 28635680 PMC5492032

[25] Gou R Gou Y Qin J Luo T Gou Q He K. Association of dietary intake of saturated fatty acids with hypertension: 1999-2018 National Health and nutrition examination survey. Front Nutr. (2022) 9:1006247. doi: 10.3389/fnut.2022.1006247, PMID: 36407507 PMC9669614

[26] Al-Abri M Al-Hashmi K Jaju D Al-Rawas O Al-Riyami B Hassan M. Gender difference in relationship of apnoea/hypopnoea index with body mass index and age in the omani population. Sultan Qaboos Univ Med J. (2011) 11:363–8. PMID: 22087378 PMC3210046

[27] Chen X Wang R Zee P Lutsey PL Javaheri S Alcantara C. Racial/ethnic differences in sleep disturbances: the multi-ethnic study of atherosclerosis (MESA). Sleep. (2015) 38:877–88. doi: 10.5665/sleep.4732, PMID: 25409106 PMC4434554

[28] Hu FB Stampfer MJ Manson JE Ascherio A Colditz GA Speizer FE. Dietary saturated fats and their food sources in relation to the risk of coronary heart disease in women. Am J Clin Nutr. (1999) 70:1001–8. doi: 10.1093/ajcn/70.6.1001, PMID: 10584044

[29] Keys A Anderson JT Grande F. Serum cholesterol response to changes in the diet: II. The effect of cholesterol in the diet. Metabolism. (1965) 14:759–65. doi: 10.1016/0026-0495(65)90002-8, PMID: 25286460

[30] Rosenthal T Oparil S. Hypertension in women. Br Med J. (2000) 14:691–704. doi: 10.1038/sj.jhh.1001095 11095160

[31] Cavallino V Rankin E Popescu A Gopang M Hale L Meliker JR. Antimony and sleep health outcomes: NHANES 2009-2016. Sleep Health. (2022) 8:373–9. doi: 10.1016/j.sleh.2022.05.005, PMID: 35753957

[32] Gaffey AE Rollman BL Burg MM. Strengthening the pillars of cardiovascular health: psychological health is a crucial component. Circulation. (2024) 149:641–3. doi: 10.1161/CIRCULATIONAHA.123.066132, PMID: 38408143 PMC10924771

[33] Levine GN Cohen BE Commodore-Mensah Y Fleury J Huffman JC Khalid U. Psychological health, well-being, and the mind-heart-body connection: a scientific statement from the American Heart Association. Circulation. (2021) 143:e763–83. doi: 10.1161/CIR.0000000000000947, PMID: 33486973

[34] Zhang Z Jackson SL Gillespie C Merritt R Yang Q. Depressive symptoms and mortality among US adults. JAMA Netw Open. (2023) 6:e2337011. doi: 10.1001/jamanetworkopen.2023.37011, PMID: 37812418 PMC10562940

[35] Grandner MA Kripke DF Naidoo N Langer RD. Relationships among dietary nutrients and subjective sleep, objective sleep, and napping in women. Sleep Med. (2010) 11:180–4. doi: 10.1016/j.sleep.2009.07.014, PMID: 20005774 PMC2819566

[36] St-Onge MP Roberts A Shechter A Choudhury AR. Fiber and saturated fat are associated with sleep arousals and slow wave sleep. J Clin Sleep Med. (2016) 12:19–24. doi: 10.5664/jcsm.5384, PMID: 26156950 PMC4702189

[37] Drozd A Kotlega D Nowacki P Ciecwiez S Trochanowski T Szczuko M. Fatty acid levels and their inflammatory metabolites are associated with the nondipping status and risk of obstructive sleep apnea syndrome in stroke patients. Biomedicines. (2022) 10:2200. doi: 10.3390/biomedicines10092200, PMID: 36140306 PMC9496373

[38] Vasquez MM Goodwin JL Drescher AA Smith TW Quan SF. Associations of dietary intake and physical activity with sleep disordered breathing in the apnea positive pressure long-term efficacy study (APPLES). J Clin Sleep Med. (2008) 4:411–8. doi: 10.5664/jcsm.27274 18853696 PMC2576325

[39] Patel K Lawson M Cheung J. Whole-food plant-based diet reduces daytime sleepiness in patients with OSA. Sleep Med. (2023) 107:327–9. doi: 10.1016/j.sleep.2023.05.007, PMID: 37285791

[40] Kohsaka A Laposky AD Ramsey KM Estrada C Joshu C Kobayashi Y. High-fat diet disrupts behavioral and molecular circadian rhythms in mice. Cell Metab. (2007) 6:414–21. doi: 10.1016/j.cmet.2007.09.006, PMID: 17983587

[41] Oosterman JE Kalsbeek A la Fleur SE Belsham DD. Impact of nutrients on circadian rhythmicity. Am J Physiol Regul Integr Comp Physiol. (2015) 308:R337–50. doi: 10.1152/ajpregu.00322.2014, PMID: 25519730 PMC4346762

[42] Micha R Mozaffarian D. Saturated fat and cardiometabolic risk factors, coronary heart disease, stroke, and diabetes: a fresh look at the evidence. Lipids. (2010) 45:893–905. doi: 10.1007/s11745-010-3393-4, PMID: 20354806 PMC2950931

[43] Hunter JE Zhang J Kris-Etherton PM. Cardiovascular disease risk of dietary stearic acid compared with trans, other saturated, and unsaturated fatty acids: a systematic review. Am J Clin Nutr. (2010) 91:46–63. doi: 10.3945/ajcn.2009.27661, PMID: 19939984

[44] Mensink RP. Effects of stearic acid on plasma lipid and lipoproteins in humans. Lipids. (2005) 40:1201–5. doi: 10.1007/s11745-005-1486-x, PMID: 16477803

[45] Sampath H Ntambi JM. The fate and intermediary metabolism of stearic acid. Lipids. (2005) 40:1187–91. doi: 10.1007/s11745-005-1484-z, PMID: 16477801

[46] Gunduz C Basoglu OK Hedner J Zou D Bonsignore MR Hein H. Obstructive sleep apnoea independently predicts lipid levels: data from the European sleep apnea database. Respirology. (2018) 23:1180–9. doi: 10.1111/resp.13372, PMID: 30133061

[47] Kruisbrink M Robertson W Ji C Miller MA Geleijnse JM Cappuccio FP. Association of sleep duration and quality with blood lipids: a systematic review and meta-analysis of prospective studies. BMJ Open. (2017) 7:e018585. doi: 10.1136/bmjopen-2017-018585, PMID: 29247105 PMC5735405

[48] Bustos-Gomez CI Gasca-Martinez D Yanez-Barrientos E Hidalgo-Figueroa S Gonzalez-Rivera ML Barragan-Galvez JC. Neuropharmacological activities of *Ceiba aesculifolia* (Kunth) Britten & Baker f (Malvaceae). Pharmaceuticals. (2022) 15:1580. doi: 10.3390/ph15121580, PMID: 36559031 PMC9785833

[49] Nakamura H Tsujiguchi H Kambayashi Y Hara A Miyagi S Yamada Y. Relationship between saturated fatty acid intake and hypertension and oxidative stress. Nutrition. (2019) 61:8–15. doi: 10.1016/j.nut.2018.10.020, PMID: 30682705

[50] Alves NF de Queiroz TM de Almeida TR Magnani M de Andrade BV. Acute treatment with Lauric acid reduces blood pressure and oxidative stress in spontaneously hypertensive rats. Basic Clin Pharmacol Toxicol. (2017) 120:348–53. doi: 10.1111/bcpt.12700, PMID: 28054477

[51] Nakatsuji T Kao MC Fang JY Zouboulis CC Zhang L Gallo RL. Antimicrobial property of lauric acid against *Propionibacterium acnes*: its therapeutic potential for inflammatory acne vulgaris. J Invest Dermatol. (2009) 129:2480–8. doi: 10.1038/jid.2009.93, PMID: 19387482 PMC2772209

[52] Mensink RP Zock PL Kester AD Katan MB. Effects of dietary fatty acids and carbohydrates on the ratio of serum total to HDL cholesterol and on serum lipids and apolipoproteins: a meta-analysis of 60 controlled trials. Am J Clin Nutr. (2003) 77:1146–55. doi: 10.1093/ajcn/77.5.1146, PMID: 12716665

[53] Haynes PR Pyfrom ES Li Y Stein C Cuddapah VA Jacobs JA. A neuron-glia lipid metabolic cycle couples daily sleep to mitochondrial homeostasis. Nat Neurosci. (2024) 27:666–78. doi: 10.1038/s41593-023-01568-1, PMID: 38360946 PMC11001586

[54] Yao J Chen Y Xu M. The critical role of short-chain fatty acids in health and disease: a subtle focus on cardiovascular disease-NLRP3 inflammasome-angiogenesis axis. Clin Immunol. (2022) 238:109013. doi: 10.1016/j.clim.2022.109013, PMID: 35436628

[55] Butler FM Utt J Mathew RO Casiano CA Montgomery S Wiafe SA. Plasma metabolomics profiles in Black and white participants of the Adventist health Study-2 cohort. BMC Med. (2023) 21:408. doi: 10.1186/s12916-023-03101-4, PMID: 37904137 PMC10617178

[56] Goedecke JH Chorell E van Jaarsveld PJ Riserus U Olsson T. Fatty acid metabolism and associations with insulin sensitivity differs between Black and white south African women. J Clin Endocrinol Metab. (2021) 106:e140–51. doi: 10.1210/clinem/dgaa696, PMID: 32995848

